# Remarkable Recovery in a Patient with Lethal Dose Paraquat Poisoning: A Case Report

**DOI:** 10.31729/jnma.8263

**Published:** 2023-09-30

**Authors:** Nishob Adhikari, Nibedita Chapagain, Rajat Acharya, Anil Pokhrel, Aliska Niroula

**Affiliations:** 1 Kathmandu Medical College and Teaching Hospital, Sinamangal, Kathmandu, Nepal; 2 Department of Internal Medicine, Kathmandu Medical College and Teaching Hospital, Sinamangal, Kathmandu, Nepal; 3 Department of Nephrology, Kathmandu Medical College and Teaching Hospital, Sinamangal, Kathmandu, Nepal

**Keywords:** *antioxidants*, *case reports*, *corrosive*, *paraquat*, *steroids*

## Abstract

Paraquat emerges as a formidable medical dilemma in Southeast Asia, its toxic effects attributed to the generation of free radicals and oxidative stress, with a specific predilection for diverse tissues, most notably the lungs. The scarcity of effective treatment modalities in resource-constrained settings magnifies the magnitude of the paraquat poisoning predicament. This report outlines the successful management of a 25-year-old man who ingested a lethal dose of paraquat. The patient presented solely with dysphagia devoid of accompanying symptoms, regardless of ingesting a fatal quantity of paraquat. The diagnosis was made based on history and a thorough clinical examination. Early, aggressive treatment with pulse therapy of steroids and antioxidants led to unexpected and quirky recovery. The case stresses the importance of prompt management and highlights the need for more research and public education to prevent future cases.

## INTRODUCTION

Paraquat poisoning presents as a significant medical concern in Southeast Asia with clinical presentation, ranging from localised gastrointestinal symptoms to severe poisoning depending on the degree of intoxication.^1,2^ Ingestion of high doses of paraquat (≥60 ml) is associated with a survival rate below 1%, often leading to pulmonary complications.^3^ Treatment options include extracorporeal elimination, intravenous antioxidants, diuresis, and supportive therapies.^4^ The effectiveness of pulse steroid therapy in reducing mortality rates remains inconclusive, despite several studies.^1^ In this context, we report a noteworthy case of severe paraquat poisoning in a young male, highlighting the efficacy of pulse steroid therapy and his intriguing recovery.

## CASE REPORT

A 25-year-old male presented at the emergency department (ED) with complaints of difficulty swallowing for 3 days. The informant gave an alleged history of "paraquat dichloride 24% SL" ingestion under the influence of alcohol. Poison was identified as paraquat on the basis of recollection by the patient and examination of the bottle brought alongside and consumed approximately 60 ml of the compound.

Within 30 min of ingestion, he arrived at a nearby hospital where gastric lavage was done and 8 mg of dexamethasone was administered intravenously. After an initial evaluation, they decided to leave against medical advice and presented to our centre after 3 days.

**Figure 1:**
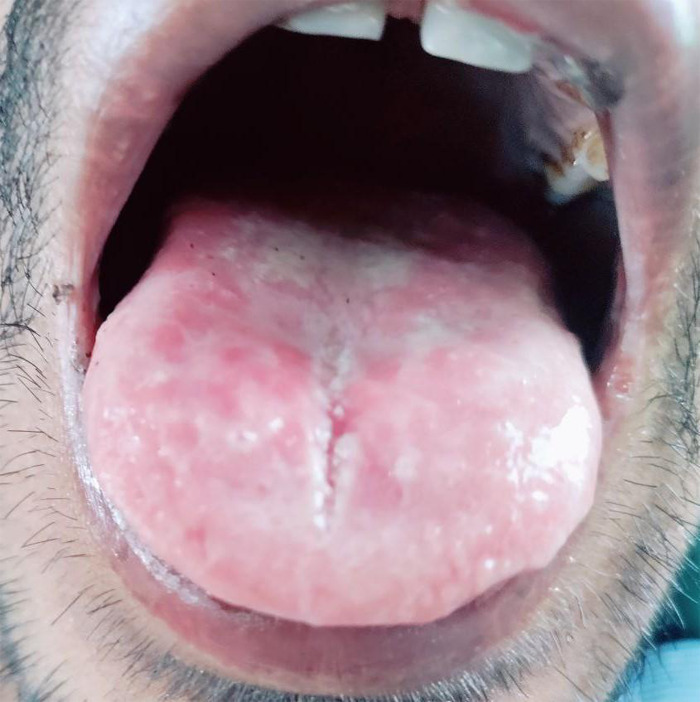
Illustrative image of the tongue of the patient suggestive of corrosive injury.

His vitals were within normal limits at presentation in the ED. His systemic examination was unremarkable. A thorough dental examination revealed extraoral manifestation in the form of hemorrhagic crustation on the lower lip and intraoral manifestation in the form of whitish plaque-like lesions on the mucosa, palate, and gingiva suggestive of corrosive injury (Figure 1).

A local anaesthetic was prescribed for this. His initial biochemical and haematological investigations were normal. Only leukocytosis was noted (total count: 12500/mm^3^, neutrophils: 90%, lymphocytes: 7%). The patient was immediately shifted to the intensive care unit (ICU) for observation and further assessment. He was kept nil per oral and vitals were monitored regularly. Pulse therapy of high dose steroid was started; intravenous (IV) methylprednisolone 1 g once daily for 5 days. He has also been prescribed antioxidants; vitamin C, acetylcysteine and zinc. IV ceftriaxone was given as a prophylactic measure. His stay at the ICU was uneventful. Chest radiographs were normal. Later IV steroid was tapered to oral dosage.

**Figure 2:**
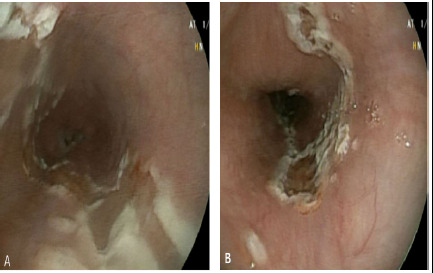
Illustrative endoscopy findings in patients of paraquat poisoning, A) Esophagus: mucosal injurysuperficial ulceration with whitish membrane seen from 30-36 cm from incisura, B) Gastroesophageal junction.

On performing an upper gastrointestinal endoscopy (UGIE), Zargar 2a pattern of corrosive injury was noted on the oesophagus (Figure 2).

Sucralfate and a proton pump inhibitor were given for symptomatic relief. The UGIE findings affirmed corrosive injury due to paraquat ingestion. Psychiatric counselling along with long-term complications of poison on the vital organs of the patient was done at the time of discharge and was advised for a follow-up in a week which was unremarkable.

## DISCUSSION

Paraquat also known as (N, N-dimethyl-4, 4'-bipyridinium dichloride; PQ) is a widely used lethal herbicide. The easy and cost-friendly availability of this compound has made prevention a major obstacle in the context of developing countries like Nepal.^4^

As an herbicide paraquat mainly affects the intracellular electron transport system. In the human body, it has a high affinity for Clara cells and types 1 and 2 pneumocytes of the lungs.^5^ The rapid distribution of paraquat in the lungs via an energy-dependent process makes it very vulnerable to damage. Paraquat, being an electron acceptor disrupts the antioxidant system of the cells. It leads to the production of a large number of reactive oxygen species (ROS). ROS leads to the activation of different pro-inflammatory protein complexes such as phosphoinositide 3-kinases (PI3K), nuclear factor-kB (NF-kB), and activator-protein 1 leading to cell growth proliferation and differentiation. The pattern of injury usually progresses from acute lung injury to lung fibrosis leading to respiratory failure and death. Paraquat can also affect the renal tubules and produce acute kidney injury in about 50% of patients.^1,4^

Paraquat has unique toxicokinetics. It is rapidly but incompletely absorbed and follows the pharmacokinetics of a two-compartment model with time-dependent elimination by the kidney from a central compartment like plasma and slow elimination from deeper compartments like lungs. This contributes to its high mortality which is as high as 90%.^1^

The clinical manifestation of PQ poisoning usually depends on the amount ingested and can be classified into three categories: In cases of mild poisoning (less than 20 mg PQ ion per kg of body weight), patients usually remain asymptomatic with minor gastrointestinal symptoms and have a good prognosis. A mucosal lesion in the form of ulceration and bleeding is more commonly seen in the oral cavity, pharynx and oesophagus. Strawberry tongue is another peculiar finding in paraquat poisoning. Severe poisoning (20-40 mg PQ ion per kg of body weight), in which patients develop acute kidney dysfunction, acute hepatic involvement in the form of jaundice and elevated transaminases, acute alveolitis and progressive pulmonary fibrosis, with death occurring up to 5 weeks later as a result of respiratory failure; and fulminant poisoning (more than 40 mg PQ ion per kg of body weight), in which patients develop renal failure, cardiac arrhythmias, coma, convulsions, and oesophagal perforation leading to death within hours to a few days after ingestion.^4,6^ The mortality rate is around 60-90% in patients with high-dose poisoning, with the lethal dose being around 20 ml.^3^ In reference to a previously conducted study, the following are identified significant prognostic factors for PQ intoxication based on the initial investigations as age, the amount of PQ ingested, plasma PQ concentration, leukocyte count, blood urea nitrogen, serum creatinine, uric acid, aspartate aminotransferase, alanine aminotransferase, and amylase. Among them, blood and urine concentration of PQ is an important predictor for prognosis.^4^ Despite its diagnostic potential, technical and financial issues limit its use. Past study has shown that the use of neutrophilic granulocyte ratio, leukocyte count, and eGFR could be an ideal early predictor of mortality in patients with acute PQ poisoning.^7^ In our case, we could assess all parameters except the plasma PQ concentration due to unavailability.

There is no specific antidote or chelating agent of PQ, so the treatment is usually supportive. The main pathological spectrum produced by PQ is the induction of ROS and production of inflammatory cytokines so, the use of high-dose glucocorticoids, cytotoxic agents and antioxidants are proven lines of management. A previous study showed that the use of high-dose long-term antioxidant therapy in the form of vitamin C, glutathione and N-acetyl cysteine significantly improved the patient's survival rate, prevented lung fibrosis in the advanced stage, and improved lung and liver function of patients.^3^ Similarly, the role of haemoperfusion and haemodialysis has been debatable.^1^ In our specific case, early gastric decontamination and the meticulous application of high-dose steroids showcased remarkable efficacy in attenuating complications and arresting deterioration of the patient's condition, while concomitantly incorporating antioxidants such as vitamin C, acetylcysteine, and zinc further contributed to a notably enhanced patient's outcome even in the face of exposure to toxic levels of PQ.

The patient's remarkable recovery was unexpected and underscored the significance of promptly initiating an aggressive anti-inflammatory regimen. The severity of PQ clinical effects, inconclusive investigations, and insufficient evidence for definitive treatment methods pose significant challenges for medical professionals. However, as demonstrated in our case, early and aggressive pulse therapy with steroids and antioxidants can aid in the patient's speedy recovery and improve their prognosis.
